# Real-Valued 2D MUSIC Algorithm Based on Modified Forward/Backward Averaging Using an Arbitrary Centrosymmetric Polarization Sensitive Array

**DOI:** 10.3390/s17102241

**Published:** 2017-09-29

**Authors:** Weijian Si, Yan Wang, Changbo Hou, Hong Wang

**Affiliations:** 1 College of Information and Communication Engineering, Harbin Engineering University, Harbin 150001, China; swj0418@263.net (W.S.); wangyan17855@hrbeu.edu.cn (Y.W.); 2 Beijing Institute of Computer Technology and Application, Beijing 100854, China; wanghong_icta@163.com

**Keywords:** 2D DOA estimation, polarization sensitive array, MUSIC, forward/backward averaging, real-valued operations, dimension reduction, Cramer-Rao bound

## Abstract

Two-dimensional multiple signal classification (MUSIC) algorithm based on polarization sensitive array (PSA) has excellent performance. However, it suffers a high computational complexity due to a multitude of complex operations. In this paper, we propose a real-valued two-dimensional MUSIC algorithm based on conjugate centrosymmetric signal model, which is applicable to arbitrary centrosymmetric polarization sensitive array. The modified forward/backward averaging, which can be applied to the PSA, is presented. Hence, the eigen-decomposition analysis process and spectrum function computation are converted into real domain, prominently reducing the computational complexity. Then, the direction-of-arrival (DOA) estimation is decoupled from the polarization parameter estimation so that the four-dimensional spectral peak search process is avoided. The theoretical computational complexity is discussed and the Cramer-Rao bound (CRB) of DOA estimation is derived in this paper. The simulation results indicate that the proposed algorithm achieves superior accuracy in DOA estimation and has low computational complexity.

## 1. Introduction

Parameter estimation is an important area in array signal processing, such as adaptive beamforming [1,2] and direction-of-arrival (DOA) estimation [3,4,5,6,7]. Especially, the two-dimensional (2D) DOA estimation based on polarization sensitive array (PSA) shows great potential [8,9,10], which has gained considerable interests. Compared with traditional scalar array (TSA), the PSA composed of electromagnetic vector sensors (EMVSs) has more inherent merits. Besides spatial domain information, polarization domain information of the electromagnetic signals can also be measured by the PSA [11,12], which can bring greater potential capacity for parameter estimation. 

During the past decades, many effective algorithms based on TSA have been extended to PSA, such as multiple signal classification (MUSIC) [13,14], estimation of signal parameters via rotational invariance techniques (ESPRIT) [15,16,17], Root-MUSIC [18,19,20], etc. With the development of research, some specific joint DOA and polarization parameter estimation algorithms for PSA have been presented, including vector cross-product based algorithms [21,22] and hyper complex MUSIC algorithms [23,24]. In almost all of the literature considering PSAs, the data model makes use of the concatenated vector of the received signal data with multi-components, resulting in the so-called “long-vector” approach [24]. An enhanced ESPRIT algorithm which improves estimation accuracy memorably and can solve the ambiguity of DOA estimation was proposed in [22]. However, this algorithm can only be applied to uniform rectangular array (URA) composed of six component electromagnetic vector sensors, leading to great restriction on the application. Moreover, the six component electromagnetic vector sensors have serious mutual coupling effect [25] and bring great economic burden. In addition, literature [26] presented an improved MUSIC algorithm based on PSA without ambiguity. It is quite applicable to the arrays with arbitrary geometries. However, the spectrum function of this algorithm is four-dimensional (4D) with respect to DOA and polarization parameters, leading to a 4D spectral peak search process. High computational load severely decelerates the parameter estimation, therefore, the reduction of computational complexity of MUSIC-based algorithm is a problem worth studying. 

Root-MUSIC algorithm is a modified MUSIC algorithm which replaces the spectral peak searching by polynomial rooting, so as to reduce the computational complexity of DOA estimation [19,20]. Nevertheless, Root-MUSIC algorithm is generally used only for uniform linear array (ULA), since it requires a complicated preprocessing for other array structures. Reducing the number of searching points can also improve computation efficiency. For MUSIC-based algorithms using PSAs, decoupling the DOA estimation from polarization parameter estimation can reduce the dimensionality of the spectral peak searching and drastically reduce the number of iterations. A MUSIC-based solution using one-dimensional spectral peak searching has been proposed in [27] base on the ULA composed of cross-dipoles. On the other hand, it is well-known that processing the data in real domain is much faster than in complex domain. In subspace-based algorithms based on TSA, by utilizing the forward/backward (FB) averaging technique, the complex covariance matrix is converted into a real-valued matrix, thus the eigenvalue decomposition (EVD) can be calculated in real domain [28,29]. However, the FB averaging cannot be applied directly to the PSAs consist of EMVSs with multiple vibrators.

In this paper, a novel real-valued MUSIC algorithm based on arbitrary centrosymmetric PSA is proposed to achieve two-dimensional DOA estimation with low computational complexity and high accuracy. The merits are reflected in the following four aspects. First of all, the proposed algorithm is applicable to an arbitrary centrosymmetric PSA, even three-dimensional array geometry. Then, the modified FB averaging which can be applied to PSA is presented in the proposed algorithm to process the data in real domain, thus achieving a large reduction of computational cost. Furthermore, through decoupling the DOA estimation from the polarization parameter estimation, the 4D MUSIC spectrum function is dimension-reduced to a 2D function depending only on the DOA parameters. Hence, a joint search of both DOA and polarization parameters is avoided, which significantly reduces the number of iterations in searching the spectral peaks. The azimuth and elevation simultaneous searches (AESS) [30] can be applied to the proposed algorithm to perform a more efficient 2D DOA peak search process. Finally, electric co-centered crossed-dipole (ECCD) pairs with lower mutual coupling effect are used in the array and it will cause less compromise on the estimation performance than most other form of vector sensors. The simulation results demonstrate that the proposed algorithm can get accurate parameter estimation with a low computational complexity. 

The remainder of this paper is organized as follows. Section 2 gives the array signal model used throughout this paper. Then, we explicitly describe the proposed algorithm in Section 3, and discuss the estimation performance and the complexity in Section 4. The simulation results are shown in Section 5 to verify the effectiveness of the proposed algorithm. Finally, the conclusion of this paper is given in Section 6. 

Notations: We use ${\left(\cdot \right)}^{T}$, ${\left(\cdot \right)}^{*}$ and ${\left(\cdot \right)}^{H}$ to represent transpose, conjugate, and conjugate transpose of matrices, respectively. Denote operators ⊗ and ⊙ as kronecker product and Khatri-Rao (KR) product, respectively. Re(·) and Im(·) are used to indicate real part and imaginary part of complex variates, respectively. E{·} denotes the expectation operator, and det(·) denotes the determinant of matrices. The non-bold letters, lower-case bold letters, and upper-case bold letters denote scalars, vectors and matrices, respectively. Especially, $I_{N}$ denotes an N×N identity matrix, and $J_{M}$ denotes an M×M anti-identity matrix with ones on its anti-diagonal and zeros elsewhere.

## 2. Array Signal Model for DOA Estimation 

### 2.1. Received Signal Model

**Figure 1:**
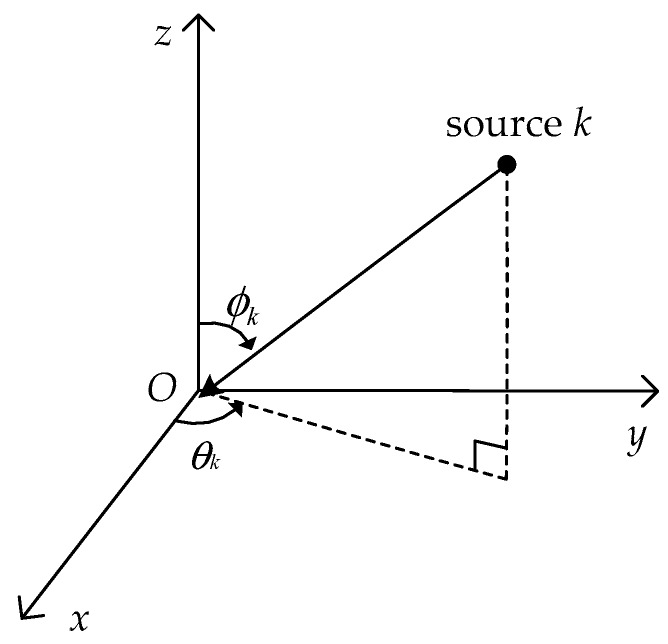
The coordinate system for the *k*-th source.

Assume that *K* uncorrelated transverse electromagnetic plane waves, which have traveled through a homogeneous isotropic medium, impinge on the electromagnetic vector sensors. Then, the *k*-th unit-power completely polarized incident source has the electric-field vector $e_{k}$ and the magnetic-filed vector $h_{k}$, which can expressed in Cartesian coordinates as [31,32,33]

(1) $$a_{p}\left(\theta_{k},\phi_{k},\gamma_{k},\eta_{k}\right)=\left[\begin{array}{c} e_{k}\\ h_{k} \end{array}\right]=\left[\begin{array}{c} e_{x,k}\\ e_{y,k}\\ e_{z,k}\\ h_{x,k}\\ h_{y,k}\\ h_{z,k} \end{array}\right]=[\begin{array}{c} -\sin\theta_{k}\;\cos\phi_{k}\cos\theta_{k}\\ \cos\theta_{k}\;\cos\phi_{k}\sin\theta_{k}\\ 0\;-\sin\phi_{k}\\ \cos\phi_{k}\cos\theta_{k}\;\sin\theta_{k}\\ \cos\phi_{k}\sin\theta_{k}\;-\cos\theta_{k}\\ -\sin\phi_{k}\;0 \end{array}]\left[\begin{array}{c} \cos\gamma_{k}\\ \sin\gamma_{k}e^{{j\eta }_{k}} \end{array}\right],$$

where $\phi_{k}\in \left[0,\pi \right]$ and $\theta_{k}\in \left[0,2\pi \right)$ denote the elevation angle measured from the vertical *z*-axis and the azimuth angle measured from the positive *x*-axis, respectively, see in Figure 1; $\gamma_{k}\in \left[0,\pi /2\right]$ and $\eta_{k}\in \left[-\pi ,\pi \right)$ denote the auxiliary polarization angle and the polarization phase difference, respectively.

Assume that a centrosymmetric polarization sensitive array consists of *M* ECCD pairs is arranged in the spatial plane of the coordinate system which includes *x*-axis, *y*-axis and the original point. For brevity, we abbreviate this plane as *xoy*-plane. The two vibrators of each ECCD pair are deployed parallel to *x*-axis and *y*-axis, respectively. Since ECCD pairs only receive components $e_{x,k}$ and $e_{y,k}$ of electric-field vector, (1) is simplified as

(2) $$a_{p}\left(\theta_{k},\phi_{k},\gamma_{k},\eta_{k}\right)=\left[\begin{array}{c} e_{x,k}\\ e_{y,k} \end{array}\right]=\underset{≜v_{p}\left(\theta_{k},\phi_{k}\right)=v_{p,k}}{\underbrace{\left[\begin{array}{cc} -\sin\theta_{k} & \cos\phi_{k}\cos\theta_{k}\\ \cos\theta_{k} & \cos\phi_{k}\sin\theta_{k} \end{array}\right]}}\underset{≜h_{r}\left(\gamma_{k},\eta_{k}\right)=h_{r,k}}{\underbrace{\left[\begin{array}{c} \cos\gamma_{k}\\ \sin\gamma_{k}e^{j\eta_{k}} \end{array}\right]}}.$$

Note that $v_{p,k}$ and $h_{r,k}$ depend only on the source spatial angular locations and the incent signal polarization states, respectively.

The spatial steering vector of the *k*-th narrow-band incent signal equals

(3) $$a_{s}\left(\theta_{k},\phi_{k}\right)={\left[e^{-j\Delta_{k1}},e^{-j\Delta_{k2}},\cdots ,e^{-j\Delta_{kM}}\right]}^{T},$$

where $\Delta_{km}$ is the inter-vector-sensor spatial phase factor relating the *k*-th source to the *m*-th vector sensor at location $\left(x_{m},y_{m},z_{m}\right)$ and is defined as

(4) $$\Delta_{km}=\frac{2\pi \left(x_{m}\sin\phi_{k}\cos\theta_{k}+y_{m}\sin\phi_{k}\sin\theta_{k}+z_{m}\cos\phi_{k}\right)}{\lambda },\;m=1,\cdots ,M,$$

where λ means wavelength. For simplicity, we use $\;a_{p,k}$ and $a_{s,k}$ to respectively represent $a_{p}\left(\theta_{k},\phi_{k},\gamma_{k},\eta_{k}\right)$ and $a_{s}\left(\theta_{k},\phi_{k}\right)$ in the following parts. As such, the received signal model (RSM) of array can be expressed as a long-vector form

(5) $$x\left(t\right)=\left(A_{s}⊙A_{P}\right)s\left(t\right)+n\left(t\right)=As\left(t\right)+n\left(t\right),$$

(6) $$A=\left[a_{1},\cdots ,a_{K}]=[a_{s,1}⊗a_{p,1},\cdots ,a_{s,K}⊗a_{p,K}\right],$$

(7) $$A_{s}=\left[a_{s,1},\cdots ,a_{s,K}\right],\;A_{p}=\left[a_{p,1},\cdots ,a_{p,K}\right],$$

(8) $$s\left(t\right)={\left[s_{1}\left(t\right),\cdots ,s_{K}\left(t\right)\right]}^{T},\;n\left(t\right)={\left[n_{1}\left(t\right),\cdots ,n_{M}\left(t\right)\right]}^{T},$$

where $n_{m}\left(t\right)$ symbolizes the 2×1 zero-mean additive complex white Gaussian noise vector at the *m*-th vector sensor.

### 2.2. Conjugate Centrosymmetric Signal Model

Suppose that there a 2M×2M permute matrix

(9) $${\tilde{P}}_{e}=P_{e}⊗I_{2},$$

where $P_{e}$ is an M×M permute matrix, and it can be obtained readily that ${\tilde{P}}_{e}{\tilde{P}}_{e}^{T}={\tilde{P}}_{e}^{T}{\tilde{P}}_{e}=I_{2M}$. Then, a novel signal model is proposed as follow

(10) $$y\left(t\right)={\tilde{P}}_{e}x\left(t\right)={\tilde{P}}_{e}\left(As\left(t\right)+N\left(t\right)\right)=A_{e}s\left(t\right)+n_{e}\left(t\right),$$

(11) $$A_{e}={\tilde{P}}_{e}A={\tilde{P}}_{e}\left(A_{s}⊙A_{P}\right)=\left({\tilde{P}}_{e}A_{s}\right)⊙A_{P}=A_{se}⊙A_{P},$$

(12) $$A_{se}={\tilde{P}}_{e}A_{s}=\left[{\tilde{P}}_{e}a_{s,1},\cdots ,{\tilde{P}}_{e}a_{s,K}\right]=\left[a_{se,1},\cdots ,a_{se,K}\right].$$

According to (3) and (4), it is obvious that the inter-vector-sensor spatial phase factors $a_{s,k}$ of two electromagnetic vector-sensors which are centrosymmetric around the origin of coordinate system are conjugate. Thus, for a centrosymmetric array, there must be a calculable and unique ${\tilde{P}}_{e}$ such that the spatial steering vector of the proposed signal model can be expressed as

(13) $$A_{se}=\{\begin{array}{cc} \left[\begin{array}{c} A_{se}^{\left(T\right)}\\ A_{se}^{\left(B\right)} \end{array}\right]=\left[\begin{array}{c} A_{se}^{\left(T\right)}\\ {\left(J_{M/2}A_{se}^{\left(T\right)}\right)}^{*} \end{array}\right], & \mathrm{for}\;\mathrm{even}\;M,\\ \left[\begin{array}{c} A_{se}^{\left(T\right)}\\ 1_{K}^{T}\\ A_{se}^{\left(B\right)} \end{array}\right]=\left[\begin{array}{c} A_{se}^{\left(T\right)}\\ 1_{K}^{T}\\ {\left(J_{\left(M-1\right)/2}A_{se}^{\left(T\right)}\right)}^{*} \end{array}\right], & \mathrm{for}\;\mathrm{odd}\;M, \end{array}$$

where $1_{K}$ denotes a K×1 matrix of ones, $A_{se}^{\left(T\right)}$ denotes the top M/2 or (M−1)/2 rows of $A_{se}$ and $A_{se}^{\left(B\right)}$ denotes the bottom part. Denote the proposed signal model as the conjugate centrosymmetric signal model (CCSM). Furthermore, note that the matrix multiplications involving ${\tilde{P}}_{e}$ or J are not counted as floating operations because they merely represent the permutations of the rows or columns of the matrix being multiplied [34].

**Figure 2:**
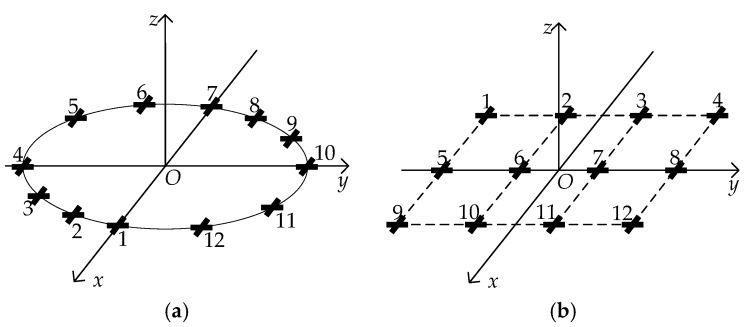
Two kinds of centrosymmetric array. (**a**) A UCA with 12 ECCDs; (**b**) A URA of 3 rows and 4 columns with totally 12 ECCDs. The numbers represent the order of vector sensors.

Without loss of generality, consider a uniform circular array (UCA) with 12 vector sensors and a uniform rectangular array (URA) with 3 rows and 4 columns which are shown in Figure 2. Consequently the permute matrix ${\tilde{P}}_{e}$ corresponding to each array is expressed as

(14) $${\tilde{P}}_{e}^{\mathrm{UCA}}=\left[\begin{array}{cc} I_{6} & 0_{6}\\ 0_{6} & J_{6} \end{array}\right]⊗I_{2},{\tilde{P}}_{e}^{\mathrm{URA}}=I_{12},$$

where $0_{6}$ denotes a 6×6 null matrix.

**Theorem** **1.** The noise-subspace and spatial steering vectors of CCSM are orthogonal.

**Proof.**  The covariance matrices of RSM and CCSM are

(15) $$R_{x}=E\left\{x\left(t\right)x^{H}\left(t\right)\right\}=U_{x}\Lambda_{x}U_{x}^{H}=U_{x,S}\Lambda_{x,S}U_{x,S}^{H}+U_{x,N}\Lambda_{x,N}U_{x,N}^{H},$$

(16) $$R_{y}=E\left\{y\left(t\right)y^{H}\left(t\right)\right\}=U_{y}\Lambda_{y}U_{y}^{H}=U_{y,S}\Lambda_{y,S}U_{y,S}^{H}+U_{y,N}\Lambda_{y,N}U_{y,N}^{H},$$

where U and Λ denote the eigenvector matrix and eigenvalue diagonal matrix, subscript *x* and *y* denote the RSM and CCSM, subscript S and N denote the signal- and noise-subspace, respectively.

According to (10) and (15), the covariance matrix of CCSM can be also expressed as

(17) $$R_{y}=E\left\{\left({\tilde{P}}_{e}x\left(t\right)\right){\left({\tilde{P}}_{e}x\left(t\right)\right)}^{H}\right\}={\tilde{P}}_{e}R_{x}{\tilde{P}}_{e}^{T}=\left({\tilde{P}}_{e}U_{x}\right)\Lambda_{x}{\left({\tilde{P}}_{e}U_{x}\right)}^{H}.$$

Compared the expressions of $R_{y}$ in (16) and (17), we have

(18) $$U_{y,S}={\tilde{P}}_{e}U_{x,S},\;U_{y,N}={\tilde{P}}_{e}U_{x,N}.$$

Note that the elements in $U_{y,S}$ and $U_{y,N}$ are just a displacement of the elements in $U_{x,S}$ and $U_{x,N}$ due to the permuted matrix ${\tilde{P}}_{e}$. Based on the MUSIC algorithm, the signal subspace is orthogonal to the noise subspace. The signal subspace is spanned by the steering vectors, which means that the steering vectors of sources are also orthogonal to the noise subspace, i.e.,

(19) $$a_{k}^{H}U_{x,N}=0,\;k=1,\cdots ,K.$$

As such, we can obtain

(20) $$a_{e,k}^{H}U_{y,N}={\left({\tilde{P}}_{e}a_{k}\right)}^{H}\left({\tilde{P}}_{e}U_{x,N}\right)=a_{k}^{H}U_{x,N}=0,\;k=1,\cdots ,K$$

which illustrates that the noise-subspace and the steering vectors of CCSM are still orthogonal. ☐

## 3. Real-valued 2D DOA Estimation Based on Modified FB Averaging

### 3.1. The Modified FB Averaging

Considering about finite received array data, the covariance matrix of CCSM is expressed as

(21) $${\hat{R}}_{y}=\frac{1}{L}\overset{L}{\underset{l=1}{\sum }}y_{l}\left(t\right)y_{l}^{H}\left(t\right)=YY^{H},$$

where *L* is the number of snapshots and $Y=1/\sqrt{L}\left[y_{1}\left(t\right),\cdots ,y_{L}\left(t\right)\right]$ is the CCSM observation matrix.

In contrast to [34,35], define the modified FB covariance matrix as

(22) $${\hat{R}}_{\mathrm{FB}}=\frac{1}{2}\left({\hat{R}}_{y}+{\tilde{J}}_{2M}{\hat{R}}_{y}^{*}{\tilde{J}}_{2M}\right)=Y_{\mathrm{FB}}Y_{\mathrm{FB}}^{H},$$

where $Y_{\mathrm{FB}}=1/\sqrt{2}\left[\begin{array}{cc} Y & {\tilde{J}}_{2M}Y^{*} \end{array}\;\right]$ is the modified FB observation matrix in which ${\tilde{J}}_{2M}$ is a 2M×2M unitary permute matrix denoted as

(23) $${\tilde{J}}_{2M}=J_{M}⊗I_{2}.$$

It is noteworthy that, different from the original FB averaging [34], the modified FB averaging uses the unitary permute matrix ${\tilde{J}}_{2M}$ conforming to (23) instead of an anti-identity matrix. This significant change avoids the confusion of the different components of the electric-field vector of the incident signals, and allows the modified FB averaging to be applied to PSAs consist of ECCD pairs. In addition, it is straightforward to certify that

(24) $${\hat{R}}_{\mathrm{FB}}={\tilde{J}}_{2M}{\hat{R}}_{\mathrm{FB}}^{*}{\tilde{J}}_{2M}$$

Then, let Q be a unitary matrix which is defined as

(25) $$Q=\{\begin{array}{cc} \frac{1}{\sqrt{2}}\left[\begin{array}{cc} I_{M} & jI_{M}\\ {\tilde{J}}_{M} & -j{\tilde{J}}_{M} \end{array}\right], & \mathrm{for}\;\mathrm{even}\;M,\\ \frac{1}{\sqrt{2}}\left[\begin{array}{ccc} I_{M-1} & 0_{\left(M-1\right)\times 2} & jI_{M-1}\\ 0_{\left(M-1\right)\times 2}^{T} & \sqrt{2}I_{2} & 0_{\left(M-1\right)\times 2}^{T}\\ {\tilde{J}}_{M-1} & 0_{\left(M-1\right)\times 2} & -j{\tilde{J}}_{M-1} \end{array}\right], & \mathrm{for}\;\mathrm{odd}\;M, \end{array}$$

where $0_{\left(M-1\right)\times 2}$ denotes an (M−1)×2 null matrix, and ${\tilde{J}}_{M-1}$ is defined similarly as (23). It is obvious that $Q={\tilde{J}}_{2M}Q^{*}$.

Therefore, we can obtain the real-valued covariance matrix as

(26) $$R_{\mathrm{RV}}=Q^{H}{\hat{R}}_{\mathrm{FB}}Q.$$

By substituting (22) into (26), $R_{\mathrm{RV}}$ can be further simplified as

(27) RRV=12(QHR^yQ+QHJ˜2MR^y*J˜2MQ)=12(QHR^yQ+QTR^y*Q*)=Re(QHR^yQ),

The second equation in (27) is derived readily by using (24). (27) indicates that $R_{\mathrm{RV}}$ only depends on the covariance of CCSM.

The real-valued covariance matrix $R_{\mathrm{RV}}$ is also symmetric due to the following properties

(28) $$R_{\mathrm{RV}}^{T}={\left(Q^{H}{\hat{R}}_{\mathrm{FB}}Q\right)}^{T}=Q^{T}{\hat{R}}_{\mathrm{FB}}^{T}Q^{*}=Q^{T}\left({\tilde{J}}_{2M}{\hat{R}}_{\mathrm{FB}}^{H}{\tilde{J}}_{2M}\right)Q^{*}=Q^{H}{\hat{R}}_{\mathrm{FB}}Q=R_{\mathrm{RV}}.$$

Thus, the EVD of $R_{\mathrm{RV}}$ require only real-valued computation [36]. Furthermore, the eigenvalues and eigenvectors of $R_{\mathrm{RV}}$ are real-valued as well.

The EVDs of ${\hat{R}}_{\mathrm{FB}}$ and $R_{\mathrm{RV}}$ are

(29) $${\hat{R}}_{\mathrm{FB}}=EΜE^{H}=E_{S}{Μ}_{S}E_{S}^{H}+E_{N}{Μ}_{N}E_{N}^{H},$$

(30) $$R_{\mathrm{RV}}=V\Pi V^{T}=V_{S}\Pi_{S}V_{S}^{T}+V_{N}\Pi_{N}V_{N}^{T},$$

where $Μ=\mathrm{diag}\left[\mu_{1},\mu_{2},\cdots ,\mu_{M}\right]$ and $\;\Pi =\mathrm{diag}\left[\pi_{1},\pi_{2},\cdots ,\pi_{M}\right]$ are eigenvalue diagonal matrices and $E=\left[e_{1},e_{2},\cdots ,e_{M}\right]$ and $V=\left[v_{1},v_{2},\cdots ,v_{M}\right]$ are eigenvector matrices. The subscript *S* and *N* represent signal- and noise-subspace, respectively.

Substituting (30) into (26), ${\hat{R}}_{\mathrm{FB}}$ can be expressed as

(31) $${\hat{R}}_{\mathrm{FB}}=QV\Pi V^{T}Q^{H}=QV_{S}\Pi_{S}V_{S}^{T}Q^{H}+QV_{N}\Pi_{N}V_{N}^{T}Q^{H}.$$

Compared (31) with (29), we have the following equations

(32) $$\Pi =Μ,\;E_{N}=QV_{N},\;E_{S}=QV_{S}.$$

Define $Z_{\mathrm{FB}}$ as

(33) $$Z_{\mathrm{FB}}=Q^{H}Y_{\mathrm{FB}}P,$$

where P is defined as

(34) $$P=\frac{1}{\sqrt{2}}\left[\begin{array}{cc} I_{L} & jI_{L}\\ I_{L} & -jI_{L} \end{array}\right].$$

Then, we can simplify $R_{\mathrm{RV}}$ by using (26), (33) and (34)

(35) $$R_{\mathrm{RV}}=Q^{H}{\hat{R}}_{\mathrm{FB}}Q=\left(Q^{H}Y_{\mathrm{FB}}P\right)\left(P^{H}Y_{\mathrm{FB}}^{H}Q\right)=Z_{\mathrm{FB}}Z_{\mathrm{FB}}^{T}.$$

Note that $Z_{FB}$ is a real-valued matrix.

Partition CCSM observation matrix as $Y=\left[\begin{array}{c} Y_{1}\\ Y_{2} \end{array}\right]\begin{array}{l} \}M\;\mathrm{rows}\;\\ \}M\;\mathrm{rows} \end{array}$ for even *M*, or $Y=\left[\begin{array}{c} Y_{1}\\ Y_{0}\\ Y_{2} \end{array}\right]\begin{array}{l} \}\left(M-1\right)\;\mathrm{rows}\\ \}2\;\mathrm{rows}\;\\ \}\left(M-1\right)\;\mathrm{rows} \end{array}$ for odd *M*. Then, substituting $Q^{H}$, P, and $Y_{\mathrm{FB}}$ yields

(36) ZFB={12[Re(Y1+J˜MY2) −Im(Y1+J˜MY2)Im(Y1−J˜MY2) Re(Y1−J˜MY2)], for even M,12[Re(Y1+J˜M−1Y2) −Im(Y1+J˜M−1Y2)2Re(Y0)   2Re(Y0)Im(Y1−J˜M−1Y2) Re(Y1−J˜M−1Y2)], for odd M.

It is easy to determine that $Z_{\mathrm{FB}}$ is always real-valued. According to the previous conclusion, the matrix multiplication involving permute matrix does not contain any floating operations, but only exchanges the corresponding rows or columns. Thus, from (36), we can deduce that calculating $Z_{\mathrm{FB}}$ does not involve general matrix multiplication, but is a simple construction based on Y (the CCSM data matrix) involving only additions. Therefore, it has significant consequences for processing modified FB covariance matrix.

### 3.2. 2D DOA Estimation with Real-Valued Spectrum Function

Applying MUSIC to the modified FB covariance matrix, the joint spatial-polarized spectrum function is

(37) $$f\left(\theta ,\phi ,\gamma ,\eta \right)=\frac{1}{a_{e}^{H}E_{N}E_{N}^{H}a_{e}}.$$

In contrast with the joint spectrum function of the customary sensor-vector MUSIC algorithm, the signal steering vectors of CCSM and the noise-subspace of the modified FB covariance matrix are used in (37). Note that calculating the noise-subspace of modified FB covariance matrix is almost a real operation, thus reducing the substantial computational amount. However, f(θ,ϕ,γ,η) is a four-dimensional function with respect to both 2D DOA parameters and 2D polarization parameters. Hence, the process of parameter estimation is actually a four-dimensional iterative peak search process which leads to large computation cost. If the four-dimensional joint spectrum function can be turned into a two-dimensional function only with respect to the 2D DOA parameters, the four-dimensional iterative search will be transformed to a two-dimensional iterative search, namely, the DOA parameters can be estimated without polarization parameters estimation. 

Substituting (7), (11) and (12) into (37), the reciprocal of the joint spectrum function equals

(38) $$g\left(\theta ,\phi ,\gamma ,\eta \right)=\frac{1}{f\left(\theta ,\phi ,\gamma ,\eta \right)}=a_{e}^{H}E_{N}E_{N}^{H}a_{e}=h_{r}^{H}H\left(\theta ,\phi \right)h_{r},$$

where

(39) $$H\left(\theta ,\phi \right)={\left(a_{se}⊗v_{p}\right)}^{H}E_{N}E_{N}^{H}\left(a_{se}⊗v_{p}\right)$$

only depends on DOA parameters, while $h_{r}$ only depends on polarization parameters. Therefore, we can obtain that

(40) $$h_{r,k}^{H}H\left(\theta_{k},\phi_{k}\right)h_{r,k}=0,$$

where $\left(\theta_{k},\phi_{k},\gamma_{k},\eta_{k}\right)$ are the DOA and polarization states of the *k*-th source, respectively.

According to the MUSIC algorithm, it is known that

(41) $$E_{N}^{H}a_{e}=E_{N}^{H}\left(a_{se,k}⊗v_{p}\right)h_{r,k}=0.$$

Furthermore, by using (39) and (41), we have

(42) $$H\left(\theta_{k},\phi_{k}\right)h_{r,k}=0.$$

It is obvious that the solution to (40) and (42) is unique, which indicates that H(θ,ϕ) is rank-deficient only when (θ,ϕ) equals the actual DOA, i.e., $\left(\theta ,\phi \right)\in {\left\{\left(\theta_{k},\phi_{k}\right)\right\}}_{k=1}^{K}$. Thus, the DOA can be estimated by solving the following optimization problem

(43) $$\left\{{\hat{\theta }}_{k},{\hat{\phi }}_{k}\right\}=\underset{\theta ,\phi }{\mathrm{argmax}}\frac{1}{\det\left(H\left(\theta ,\phi \right)\right)}.$$

Therefore, the direction parameter estimation is successfully decoupled from the polarization estimation, thus reducing the number of variables that need to be optimized.

Then, exchange the expression of H(θ,ϕ) in order to estimate the DOAs only through real-valued operations. Substituting (13) and (32) into (39), it can be obtained that

(44) $$H\left(\theta ,\phi \right)=D^{T}\left(\theta ,\phi \right)V_{N}V_{N}^{T}D\left(\theta ,\phi \right),$$

where

(45) D(θ,ϕ)=QHase⊗vp={2[Re(ase(T))Im(ase(B))]⊗vp,for even M,2[Re(ase(T))1Im(ase(B))]⊗vp,for odd M.

Obviously, H(θ,ϕ) is a real-valued matrix since D(θ,ϕ) and $V_{N}$ is real-valued. From (39) and (44), it is clear that these two forms of H(θ,ϕ) are exactly identical. However, calculating H(θ,ϕ) according to (44) only involves the operations between real-valued matrices. Therefore, (44) is a much better choice to calculate the spectrum function and estimate the azimuth angle and the elevation angle.

The whole process of the proposed algorithm is summarized in Table 1.

## 4. Discussion

### 4.1. Cramer-Rao Bound (CRB) Analysis

Cramer-Rao bound (CRB) is an important parameter in discussing the performance of a system [37]. In this subsection, the CRB of 2D DOA estimation is discussed. The array received signal model is shown in Equation (5).

The Fisher information matrix (FIM) with respect to azimuth angle $\theta =\left[\theta_{1},\cdots ,\theta_{K}\right]$, elevation angle $\phi =\left[\phi_{1},\cdots ,\phi_{K}\right]$, auxiliary polarization angle $\gamma =\left[\gamma_{1},\cdots ,\gamma_{K}\right]$ and polarization phase difference $\eta =\left[\eta_{1},\cdots ,\eta_{K}\right]$ is written as

(46) $$F=\left[\begin{array}{cc} \begin{array}{cc} F_{\theta \theta } & F_{\theta \phi }\\ F_{\phi \theta } & F_{\phi \phi } \end{array} & \begin{array}{cc} F_{\theta \gamma } & F_{\theta \eta }\\ F_{\phi \gamma } & F_{\phi \eta } \end{array}\\ \begin{array}{cc} F_{\gamma \theta } & F_{\gamma \phi }\\ F_{\eta \theta } & F_{\eta \phi } \end{array} & \begin{array}{cc} F_{\gamma \gamma } & F_{\gamma \eta }\\ F_{\eta \gamma } & F_{\eta \eta } \end{array} \end{array}\right].$$

Here, the CRB of 2D DOA estimation is solely considered. Thus, the sub-matrix of FIM with respect to azimuth and elevation is expressed as

(47) $$F_{\mathrm{DOA}}=\left[\begin{array}{cc} F_{\theta \theta } & F_{\theta \phi }\\ F_{\phi \theta } & F_{\phi \phi } \end{array}\right].$$

According to literatures [38,39], the (*i,j*)-th element of $F_{\theta \theta }$ is given by

(48) Fθθ(i,j)=L·tr{Rx−1∂Rx∂θiRx−1∂Rx∂θj}=2L·tr{A¯θiRsAHRx−1ARsA¯θjHRx−1+A¯θiRsAHRx−1A¯θjHRsAHRx−1}=2L·Re{eiTRsAHRx−1ARsejejTA¯θHRx−1A¯θei+eiTRsAHRx−1A¯θejejTRsAHRx−1A¯θei}.

Hence, the sub-matrix of $F_{\mathrm{DOA}}$ with respect to azimuth is

(49) Fθθ=2L·Re{(RsAHRx−1ARs)⊙(A¯θHRx−1A¯θ)T+(RsAHRx−1A¯θ)⊙(RsAHRx−1A¯θ)}.

Similarly, the sub-matrices with respect to elevation and the cross terms of $F_{\mathrm{DOA}}$ can be expressed as

(50) Fϕϕ=2L·Re{(RsAHRx−1ARs)⊙(A¯ϕHRx−1A¯ϕ)T+(RsAHRx−1A¯ϕ)⊙(RsAHRx−1A¯ϕ)},

(51) Fθϕ=2L·Re{(RsAHRx−1ARs)⊙(A¯ϕHRx−1A¯θ)T+(RsAHRx−1A¯ϕ)⊙(RsAHRx−1A¯θ)},

(52) Fϕθ=2L·Re{(RsAHRx−1ARs)⊙(A¯θHRx−1A¯ϕ)T+(RsAHRx−1A¯θ)⊙(RsAHRx−1A¯ϕ)}

where tr{⋅} denotes the trace of a matrix, $e_{i}$ denotes the *i*-th column of the identity matrix. $R_{s}$ is the covariance matrix of sources which is defined as $R_{s}=E\left\{s\left(t\right)s^{H}\left(t\right)\right\}$. ${\bar{A}}_{\theta }=\left[\frac{\partial A}{\partial \theta_{1}},\cdots ,\frac{\partial A}{\partial \theta_{K}}\right]$ and ${\bar{A}}_{\phi }=\left[\frac{\partial A}{\partial \phi_{1}},\cdots ,\frac{\partial A}{\partial \phi_{K}}\right]$ are the derivations of A with respect to $\theta_{i}$ and $\phi_{j}$ with i,j=1,2,⋯,K.

Then, the CRB matrix $C_{\mathrm{DOA}}$ with respect to azimuth and elevation is the inverse of $F_{\mathrm{DOA}}$, i.e.,

(53) $$C_{\mathrm{DOA}}=F_{\mathrm{DOA}}^{-1}.$$

As such, the CRBs of azimuth and elevation of the *k*-th source are

(54) $${\mathrm{CRB}}_{\theta_{k}}=C_{\mathrm{DOA}}\left(k,k\right),$$

(55) $${\mathrm{CRB}}_{\phi_{k}}=C_{\mathrm{DOA}}\left(K+k,K+k\right),$$

where $C_{\mathrm{DOA}}\left(k,k\right)$ denotes the (*k,k*)-th element of $C_{\mathrm{DOA}}$.

### 4.2. Theoretical Computational Complexity Analysis

To illustrate the computational effectiveness of the proposed algorithm, the computational complexity is explicitly discussed in this subsection comparing with LV-MUSIC algorithm [24]. The LV-MUSIC algorithm is an improved MUSIC algorithm making use of long-vector data form. It has the advantages of high resolution and superior accuracy, and can be applied to the PSAs with arbitrary structure composed of vector sensors. In view of the fairness of comparison, when considering of LV-MUSIC algorithm, assume that the polarization parameters are priori or have been estimated accurately, so that the search dimensions of the two algorithms are consistent. We abbreviate the LV-MUSIC algorithm with 2D DOAs peak searching as 2D LV-MUSIC temporarily.

The computational complexity of the proposed algorithm mainly includes the following three parts. (1) Computing the real-valued covariance matrix $R_{\mathrm{RV}}$ from RSM requires about $\left(16L-4\right)M^{2}+6LM$ flops; (2) The real-valued EVD (based on symmetric QR algorithm [40]) of $R_{\mathrm{RV}}$ requires about $72M^{3}$ flops; (3) Computing the spectrum function in (44) for each searching point requires about $16M^{3}+\left(28-8K\right)M^{2}+9M+8$ flops. Assume that there are $N_{SP}$ searching points in the interested search space. Thus, the computational complexity of the proposed algorithm is

(56) $$C_{\mathrm{proposed}}=72M^{3}+\left(16L-4\right)M^{2}+6LM+N_{SP}\left(16M^{3}+\left(12-8K\right)M^{2}+29M+4\right).$$

Similarly, the computational complexity of 2D LV-MUSIC algorithm mainly consists of following three parts. (1) Computing complex covariance matrix from RSM requires about $8\left(3L^{2}+L-1\right)M^{2}$ flops; (2) The EVD of complex covariance matrix requires about $288M^{3}$ flops; (3) Computing the spectrum function for each searching point requires about $64M^{3}+\left(56-43K\right)M^{2}+88M+21$ flops. Under the assumption that $N_{SP}$ searching points are used in the interested search space, we can obtain the computational complexity of 2D LV-MUSIC algorithm is

(57) $$C_{\mathrm{LV}-\mathrm{MUSIC}}=288M^{3}+8\left(3L^{2}+L-1\right)M^{2}+N_{SP}\left(64M^{3}+\left(56-32K\right)M^{2}+88M+21\right).$$

**Figure 3:**
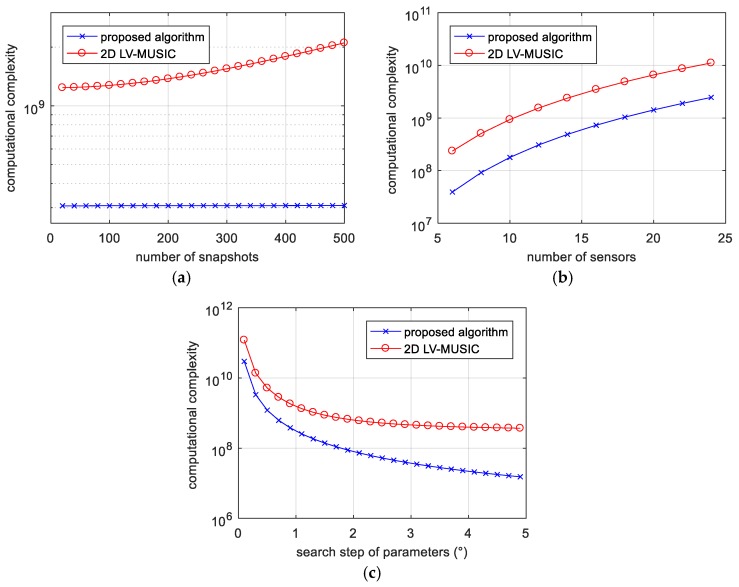
Computational complexity versus (**a**) the number of snapshots (for 12 sensors and 1° search step); (**b**) the number of sensors (for 300 snapshots and 1° search step), and (**c**) the search step of parameters (for 12 sensors and 300 snapshots).

Figure 3 shows the complexity of the proposed algorithm and 2D LV-MUSIC algorithm versus the number of snapshots, the number of sensors and the search step of parameters. From Figure 3a–c, we can find that the theoretical computational cost of both the algorithms increases as the number of snapshot and the number of the sensors become larger, while reduces as the search step of the parameters decreases. In addition, it is obvious that the computational complexity of the proposed algorithm is always lower than 2D LV-MUSIC algorithm.

## 5. Simulation Results

In this section, several simulation results are presented to illustrate the performance of the proposed algorithm. A UCA and a URA as shown in Figure 2 are used in the following simulations. The radius of UCA is 2λ and the interspace of URA is λ. Two uncorrelated far-field narrowband signals whose DOAs and polarization parameters (θ,ϕ,γ,η) are $\left(50^{{}^{\circ}},10^{{}^{\circ}},40^{{}^{\circ}},\;-120^{{}^{\circ}}\right)$ and $\left(100^{{}^{\circ}},20^{{}^{\circ}},45^{{}^{\circ}},70^{{}^{\circ}}\right)$, respectively, are assumed to impinge on the array. The first source is left-circularly elliptically polarized, whereas the second source is right-circularly elliptically polarized. Complex additive Gaussian white noise is added into the system.

The root mean square error (RMSE) of the *k*-th source is defined as

(58) $${\mathrm{RMSE}}_{k}=\sqrt{\frac{1}{N}\overset{N}{\underset{n=1}{\sum }}{\left({\hat{\varsigma }}_{k,n}-\varsigma_{k}\right)}^{2}},\;\varsigma =\;\theta ,\phi ,$$

where N is the number of independent Monte Carlo trails. $\varsigma_{k}$ is the parameter of the *k*-th source, and ${\hat{\varsigma }}_{k,n}$ is the estimated values of $\varsigma_{k}$ in the *n*-th trail.

### 5.1. The Simulation Results Distribution Scatter Plots

**Figure 4:**
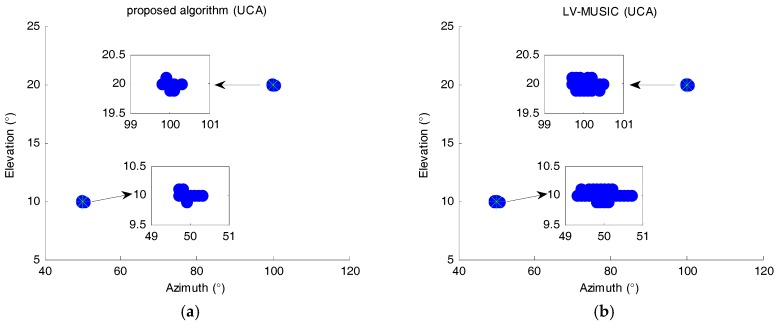
Scatter plots of 2D DOA estimation for (**a**) the proposed algorithm and (**b**) 2D LV-MUSIC algorithm.

Figure 4 shows the simulation results of the elevation and the azimuth of the proposed algorithm and 2D LV-MUSIC algorithm in 200 trials. The signal-noise ratio (SNR) is 10 dB, and the number of snapshots is 300. The search step is set as 0.1°. The green asterisk symbols represent the actual DOA values, while the blue points represent the estimated DOA values. The small windows inside each figure are the enlarged version of the dotted areas. In Figure 4, the estimated DOA values always cluster around the actual DOA values, which demonstrate that both two algorithms derive a small estimation bias and variance. Note that in Figure 4, all the simulation results are distributed on the searching points, which is a characteristic of the spectral peak search-based algorithms. In addition, from Figure 4a, it can be seen that the maximum evaluated error of elevation and azimuth angles of proposed algorithm are less than 0.2° and 0.5°, respectively; from Figure 4b, we can see that the maximum evaluated error of elevation and azimuth angles of the 2D LV-MUSIC algorithm are less than 0.2° and 0.9°, respectively. That is, the scatter plots of proposed algorithm concentrate on a smaller area than 2D LV-MUSIC algorithm, which manifests that the proposed algorithm has a better estimation performance.

### 5.2. The Estimation Performance versus SNR and the Number of Snapshots

In this subsection, the RMSEs of the proposed algorithm and 2D LV-MUSIC algorithm versus SNR and the number of snapshots are compared. The azimuth angle and elevation angle are searched in the range of 0° to 360° and 0° to 90° with a step of 0.1°, respectively. 500 Monte Carlo trails are performed to acquire the RMSE. 

**Figure 5:**
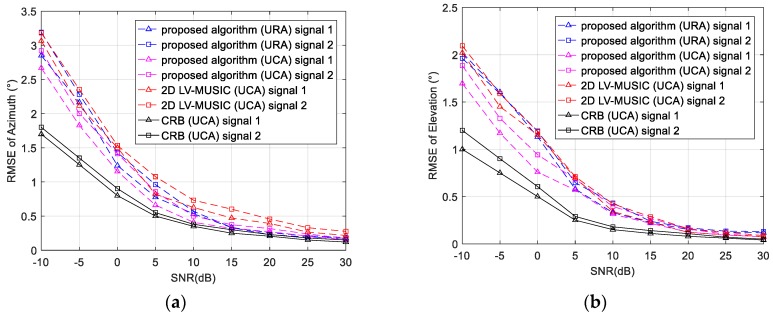
RMSE and CRB of (**a**) azimuth and (**b**) elevation versus SNR (for 300 snapshots).

We first examine the RMSE versus SNR where the SNR of the signals varies from −10 dB to 30 dB with a step of 5 dB and the number of snapshots is set as 300. As shown in Figure 5, it is obvious that for the estimation of the azimuth, the proposed algorithm can achieve a lower RMSE than the 2D LV-MUSIC especially for the UCA. As to the estimation of the elevation, in low SNR region, the proposed algorithm using UCA outperforms the 2D LV-MUSIC. In high SNR region, the performance of the proposed algorithm and the 2D LV-MUSIC is similar for estimating the elevation. However, as analyzed in Section 4.2, the computational complexity of the proposed algorithm is much lower than the 2D LV-MUSIC. 

**Figure 6:**
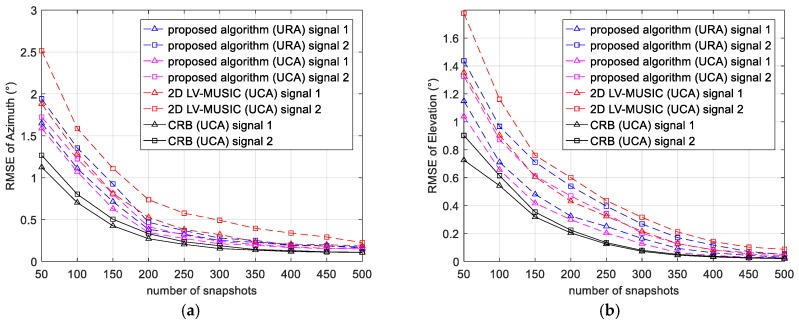
RMSE and CRB of (**a**) azimuth and (**b**) elevation versus number of snapshots (for SNR = 10 dB).

Then, we will examine the DOA estimation performance of the proposed algorithm and 2D LV-MUSIC algorithm versus number of snapshots by varying the number of snapshots from 50 to 500 with a step of 50. The SNR is set as 10 dB. The simulation results are plotted in Figure 6. We can easily find that for the estimation of both azimuth and elevation, the proposed algorithm can get a better estimation result than the 2D LV-MUSIC. 

Figure 5 and Figure 6 verified the effectiveness of the proposed algorithm. This is mainly because when the modified FB observation matrix is obtained by the CCSM observation matrix, the size of the data matrix yields an effective doubling, which achieves an expected improvement in the estimator variances. The improvement makes the proposed algorithm have preferable robustness and higher estimation accuracy of direction finding. Furthermore, a better estimation performance of the proposed algorithm can be obtained when UCA is used.

### 5.3. Running Time Comparison

The runtime of the proposed algorithm and LV-MUSIC algorithm under the same conditions is compared in this subsection. The simulation results are obtained using a PC with an Inter(R) Pentium(R) G2010 2.8 GHz CPU and 8 GB RAM by running the Matlab (Ver. R2016b) codes in the same environment. Two signals are considered here. 200 Monte Carlo trails are carried out with 10 dB SNR and 300 snapshots. The azimuth angle and the elevation angle have been searched in the range of 0° to 360° and 0° to 90°, respectively. The average running time with different numbers of sensors and different search steps are shown in Table 2. It can be seen that the running time of the proposed algorithm is lower than the 2D LV-MUSIC, namely, the proposed algorithm is more efficient than the 2D LV-MUSIC in computational complexity. This is mainly because the proposed algorithm transforms the computation of the spectrum function from the complex space into the real-valued space, so that the computing time is significantly reduced at each searching point. It is worth noting that the number of searching points in the LV-MUSIC algorithm with 4D search is several orders of magnitude larger than that of the algorithm with 2D search. Thus, its running time is unacceptable, and it is not considered as a comparison here. 

## 6. Conclusions

This paper proposes a real-valued 2D MUSIC algorithm based on CCSM data matrix, which ensures high computational efficiency and can be applied to arbitrary centrosymmetric PSA. Due to the modified FB averaging presented, the complex covariance matrix and spectrum function are converted into real domain. As a result, the computational cost is reduced significantly. By utilizing the rank deficiency theorem, the spectrum function which only depends on the DOA parameters is obtained. As such, the 2D azimuth and elevation can be estimated without estimating polarization parameters. Furthermore, in practical implementation, the search space and search step can be set reasonably under different circumstances in order to minimize the time of DOA estimation with the satisfaction of estimation accuracy. Taking UCA and URA for examples, the DOA estimation performance and computational complexity are discussed by several simulations. The simulation results validate that the proposed algorithm outperforms the 2D LV-MUSIC algorithm and has a lower running time. 

## Figures and Tables

**Table 1:** Implementation steps of the entire approach.

Input	The Received Signal Model (RSM) of Array $x\left(t\right)=\left(A_{s}⊙A_{P}\right)s\left(t\right)+n\left(t\right)$.
Output	2D DOA Estimation.
Step 1	Obtain the CCSM observation matrix $Y=1/\sqrt{L}\left[y_{1}\left(t\right),\cdots ,y_{L}\left(t\right)\right]$.
Step 2	Compute the real-valued matrix $Z_{\mathrm{FB}}$ by (36).
Step 3	Compute the symmetric real-valued covariance matrix $R_{\mathrm{RV}}=Z_{\mathrm{FB}}Z_{\mathrm{FB}}^{T}$.
Step 4	Perform the EVD of $R_{\mathrm{RV}}$ by (30) to get $V_{N}$.
Step 5	Compute the spectrum function at each searching point via $H\left(\theta ,\phi \right)=D^{T}\left(\theta ,\phi \right)V_{N}V_{N}^{T}D\left(\theta ,\phi \right)$, and estimate 2D azimuth angle and elevation angle of *K* signals by $\left\{{\hat{\theta }}_{k},{\hat{\phi }}_{k}\right\}=\mathrm{argmax}1/\det\left(H\left(\theta ,\phi \right)\right)$.

**Table 2:** Comparison of the average running time (s) with 200 Monte Carlo trails.

Search Step	Number of Sensors	Proposed Algorithm	LV-MUSIC (2D Search)
0.25	12	17.6096	21.4674
0.5	12	4.8517	5.5413
1	12	1.1539	1.4175
0.5	6	3.9773	4.5010
0.5	18	6.2451	8.5407
